# Abstract Representation of Movement Parameters in Basal Ganglia Output

**DOI:** 10.1002/advs.77621

**Published:** 2026-09-14

**Authors:** Ana Silvia Báez‐Cordero, Diana I. Ortega‐Romero, Claudia I. Pérez‐Díaz, Pavel E. Rueda‐Orozco

**Affiliations:** ^1^ Department of Neurobiology of Development and Neurophysiology Institute of Neurobiology UNAM Juriquilla Mexico

**Keywords:** basal ganglia, computer science, forelimb, kinematics, neuroscience, optogenetics, scaling, signal

## Abstract

An attractive hypothesis is that the basal ganglia (BG) are implicated in controlling and adapting movement kinematics to different behavioral contexts. We explored this possibility by recording *substantia nigra pars reticulata* (SNr) spiking activity in freely moving rats during movement execution using two behavioral protocols with different spatiotemporal content. First, animals were trained to perform locomotion runs constrained to a spatiotemporal range of meters and a few seconds (∼7 s). Then, the same animals were trained to perform forelimb movements restricted to 3–10 cm in the order of hundreds of milliseconds. We found that the spiking activity of SNr neurons recorded in both tasks was mainly and linearly correlated with movement position and velocity, with these representations scaling across different spatiotemporal contexts. That is, the minimum and maximum firing rates were proportionally adjusted to the corresponding ranges of velocity/position in each task. Finally, execution representations, their scaling properties, and the behavioral effects of pathway‐specific optogenetic manipulations were similar across SNr subpopulations. Our data suggest that the SNr integrates upstream information into a common, scalable velocity/position output signal that is broadcast to multiple target structures, supporting the idea that the BG contribute to the abstract representation of movement kinematic control.

## Graphical abstract

The basal ganglia output provides a scalable velocity/position signal across behavioral contexts, supporting the idea that these variables are represented in the brain as abstract entities rather than as fixed changes in joint angles or specific sets of body effectors.

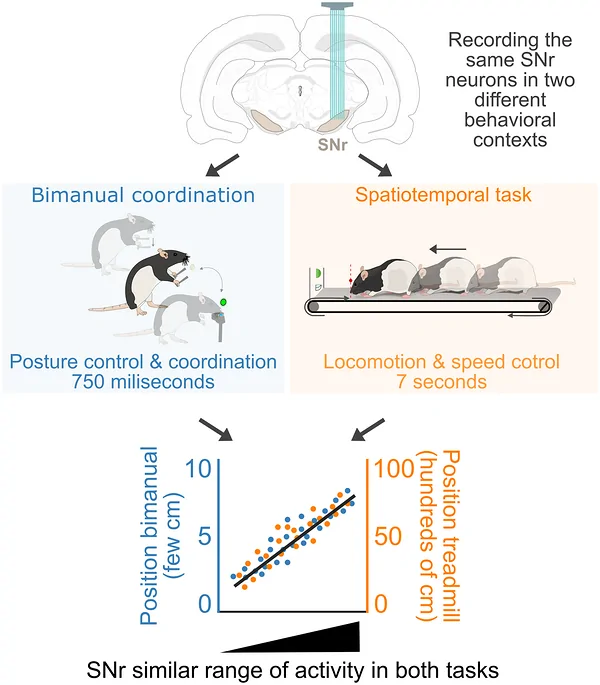

## 1 Introduction

Behavior is a continuous sequence of actions unfolding over time. Behavioral control involves integrated dynamics between the sensory and motor cortical and subcortical neural circuits spread throughout the nervous system, including the basal ganglia (BG) [1, 2]. Researchers have proposed that the BG are subcortical nuclei implicated in action selection [3, 4] and habit formation [5, 6]. They have also been shown to play a crucial role in the modulation of movement parameters, such as timing, vigor, amplitude, and speed [1, 7, 8, 9, 10]. Previous literature has attempted to reconcile these diverse functions with the classical perspective [11] of opposing and segregated direct/indirect BG pathways arising from the striatum and converging on the BG output nuclei, the internal segment of the *globus pallidus* and the *substantia nigra pars reticulata* (SNr). This perspective predicts that activating (inactivating) the direct (indirect) pathway would reduce (increase) the activity of the SNr, pausing (enhancing) the tonic inhibition exerted on motor thalamic and mesencephalic structures and promoting (inhibiting) movement [11, 12]. Research supports this viewpoint by demonstrating that locomotion control results from bulk optogenetic activation or inactivation of the striatal origin of these pathways [13, 14]. It has also been proposed that pharmacologically induced unbalanced direct/indirect pathway integration in the SNr causes abnormal control of movement speed [15].

Together with these manipulative approaches, previous studies have shown that striatal spiking activity is associated with a variety of events and parameters, including transient activations at movement initiation and termination [4, 16, 17], representations of speed, position, and timing of movement sequences [7, 8, 9, 18, 19, 20], and representations of self‐velocity and distance during pursuit behavior [21]. These observations further support that the BG is involved in both moment‐to‐moment modulation of movement parameters and action selection. However, how do these striatal signals reach the output nuclei of the BG and influence behavior? SNr activity has been shown to exhibit both higher and decreased firing rates in response to the optogenetic activation of direct and indirect pathways. However, movement inhibition and promotion were associated with discrete SNr subpopulations that appear to follow the classical model [22]. Consistent with this view, studies have also reported that cancelling and slowing down the onset of short displace movements depends on timed signal integration of the direct/indirect pathways in the spiking activity of the SNr [23, 24]. Importantly, it has also been shown that SNr spiking activity linearly represents instantaneous components of spontaneous posture [25] and body position coordinates [25], and that modulated optogenetic striatonigral activation linearly modulates velocity in rodents [26]. While the literature suggests that the BG may be involved in the continuous representation of movement execution variables, such as velocity or position, the functional importance of the output is still far from being completely understood.

The previous research addressed the role of the SNr in controlled and reduced movement conditions, but recent studies in rodents have shown a much more complex scenario, with topographically and functionally specialized subpopulations throughout the BG, including the SNr. Their findings reveal a correspondence between circuit anatomy and function for supporting behavioral specificity. Activating these subpopulations produces differential behavioral patterns, such as direction‐specific turns, licking, grooming, or reaching [27, 28, 29, 30]. Moreover, behavioral control also involves the adjustment of multiple movement execution parameters (e.g., timing, velocity, or amplitude) to different spatiotemporal contexts and scales. In motor cortical and striatal regions, codes for movement timing, velocity, or amplitude have been shown to adapt or scale [8, 31, 32, 33, 34]. But little is known about the permeation of these adaptive features from a significantly larger and complex microstructure, such as the dorsolateral striatum (DLS; about 3 of millions of neurons, with interneurons, in rodents), to the interneuron‐less microstructure of the output nuclei of the BG, which consists of only a few dozen thousand neurons [35]. Here, we performed high‐density electrophysiological recordings in freely moving rats executing two different behavioral protocols to explore the possibility that the activity of the SNr adapts its neuronal representations of different movement execution parameters to behavioral contexts with distinct spatiotemporal and cognitive demands. We found that SNr activity was correlated with the kinematic parameters of execution, especially with position and velocity. Furthermore, these kinematic representations were adjusted to the spatiotemporal movement scales. However, the same neurons recorded in both tasks demonstrated heterogeneous subpopulations maintaining or changing their functional identity across tasks. Next, by performing optogenetic antidromic identification, we found that SNr neurons could be divided into subpopulations projecting to the ventrolateral and ventromedial motor thalamus (VL/VM), the cuneiform nucleus (CnF), or both, but importantly, these subpopulations expressed similar encoding and scaling values, confirming a common output signal. Our data suggest that the BG, and in this case the SNr, represent movement parameters as “abstract entities” broadcast to multiple anatomical targets. These movement parameters can be generalized to distinct movements and contexts and are not specific to articulations or joints. This type of neural code has been proposed as a general mechanism to produce motor plans typically allocated in the motor and premotor cortices.

## 2 Results

### 2.1 SNr Hosts Movement Representations in a Locomotion‐Based Protocol

Previous reports have shown robust kinematic and contextual representations in the BG (striatum) input in tasks involving various spatiotemporal action scales [7, 18]. Hence, our first objective was to determine if those representations would carry over to the BG output, the SNr. We aimed to record SNr neural activity in freely behaving rats in two different behavioral protocols. In our first protocol, we trained rats to obtain rewards according to a spatiotemporal rule by executing a controlled sequence of accelerations and decelerations. In this task, behavioral content was executed in a range of 2–20 s, and the animals moved in a spatial range of hundreds of centimeters. We used a slightly modified version of the previously described treadmill‐based spatiotemporal task [7, 9]. During the first phase of training, the treadmill belt moved at a fixed speed, and animals learned to avoid the front of the treadmill (goal area) for at least 7 s (goal time) before performing a controlled acceleration across the apparatus. Correct trials (entering the goal area after the goal time) were rewarded by switching off the treadmill and delivering a drop of sucrose water into a water well on the apparatus's front wall. Incorrect trials (entering the goal area before the goal time) were not rewarded; instead, they were signaled with an auditory cue (1.5 kHz), and depending on the magnitude of the error, the animals were punished with an additional 13–18 s run. This phase lasted about 30–50 sessions. Then, to understand how speed control contributes to the general architecture of movement sequences, we implemented a second phase of training. During each session, the treadmill speed varied randomly from trial to trial, so the animals were forced to dynamically adapt their speed based on their perception of the treadmill's speed. Seven speeds ranging from 27 to 33 cm/s, were randomly presented on a trial‐by‐trial basis. Animals were trained in this version of the task for at least 30 sessions.

**FIGURE 1:**
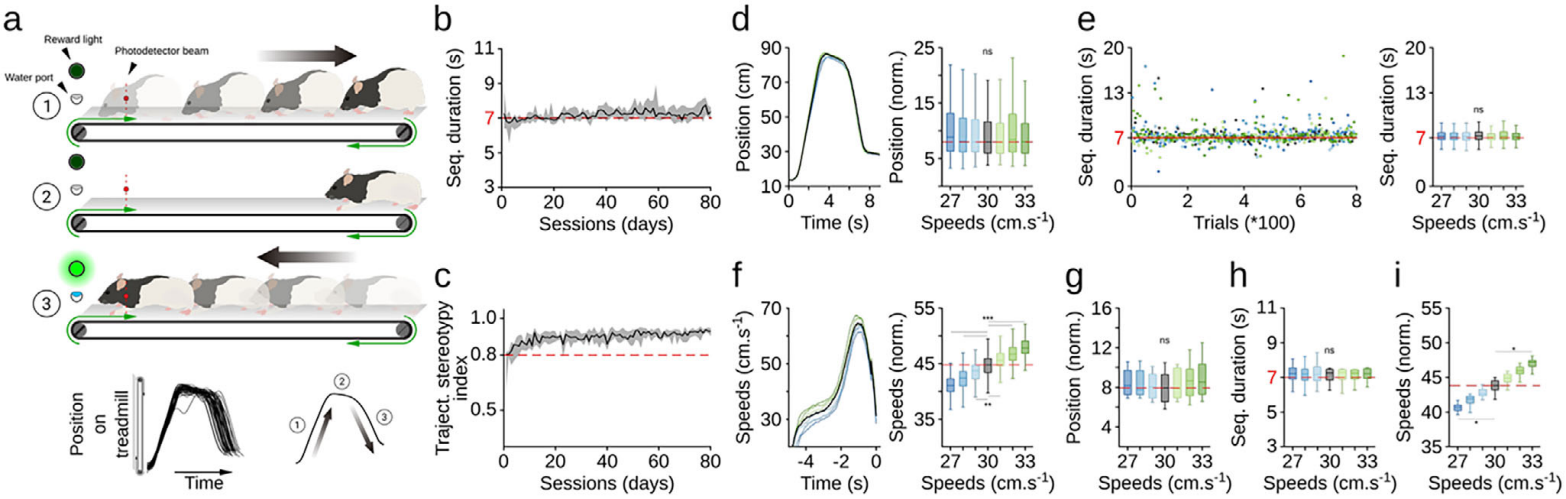
Spatiotemporal behavioral protocol. (a) Schematic representation of the different phases of the Front–Back–Front strategy expressed by the animals in the spatiotemporal task. Trajectories for every trial and the average trajectory of a representative session are presented at the bottom. The different phases of the sequence are indicated in the average trajectory. Sequence duration (b) and trajectory stereotypy index (c) are expressed as median (solid line) + 75th and 25th percentiles (shaded area) for the entire learning curve. (d) Representative average position trajectories for a representative, well‐trained animal on trials at different treadmill speeds (color‐coded, left) and trajectory differences with respect to the stereotypical position trajectory at the center treadmill speed (right, boxplots). (e) Sequence durations for individual trials (left) and groups of trials (right) at different treadmill speeds (color‐coded dots and boxes). (f) Same as in d but for the animal's speed trajectories. Data in d–f correspond to >600 trials of a representative animal. Group data (*n* = 6) for position (g), sequence duration (h), and speed (i). The central line and box in boxplots (d–i) represent the median and 25th and 75th percentiles. Whiskers extend to the most extreme data points excluding outliers. Bonferroni *post hoc* test *** *p* < 0.05.

Well‐trained animals typically executed locomotion sequences in a “Front–Back–Front” (F–B–F) pattern, which included a passive move from the front to the back of the treadmill, a “holding period” during which the animals stayed in place by running at the treadmill speed, and then a final acceleration across the treadmill to return to the front area where they reached their peak speed (Figure 1a). After extensive training, the animals executed F–B–F sequences with low spatiotemporal variability (Figure 1a, bottom) and close to the goal time of 7 s (Figure 1b). Furthermore, F–B–F sequences reached high levels of stereotypy, with great trajectory similarity between trials (Figure 1c).

During the second phase of training, we changed the treadmill's speed randomly on a trial‐by‐trial basis, but the animals maintained the general architecture of the F–B–F sequence regardless of the treadmill speed, suggesting a skillful adaptation to this variable (Figure 1d, left; overlapped average trajectories for each treadmill speed, color‐coded). To formally quantify this possibility, we calculated the absolute difference between each trial trajectory and the average trajectory of the trials performed at the central speed (30 cm s^−1^). Then, the trajectory differences corresponding to each treadmill speed were grouped and compared statistically. The differences in trajectories between individuals and the group of animals were not statistically significant (Figure 1d, right; Kruskal–Wallis [K–W], *df* = *6; X^2^* = *5.91; p* = *0.43*). This means that the animals were able to keep their spatiotemporal architecture even when they were performing the movement sequences at different speeds. This possibility was further confirmed by analyzing the sequence durations and verifying that the changes in treadmill speed did not significantly affect them (Figure 1e; K–W, *df* = *6; X^2^* = *3.59; p* = *0.73*). Finally, upon analyzing the peak locomotion speed when crossing the treadmill during the last phase of the sequence, we found that the animals linearly adjusted their own speed to match the current treadmill speed. That is, when the treadmill was running at lower and higher speeds, animals produced slower or faster accelerations, respectively (Figure 1f; K–W, *df* = *6; X^2^* = *739.08; p* < *0.001 o p* = *2.22e‐156*). These three variables (position on the treadmill, sequence duration, and peak speed) behaved similarly in individual animals and in the group of animals (Figure 1g–i; *n* = 6; G, K–W, *df* = *6, X^2^* = *2.09, p* = *0.91;* H, K–W, *df* = *6, X^2^* = *0.24, p* = *0.99;* I, K–W, *df* = *6, X^2^* = *74.42, p* = *5.02e‐14*).

The previous behavioral data indicate that the rats efficiently adapted their speed to the contextual demands to maintain a fixed, highly trained motor program characterized by a stereotypical spatiotemporal architecture. Previous reports indicate that the DLS, one of the main input structures of the BG, hosts linear representations of movement parameters, such as locomotion speed or position on the treadmill, while animals execute stereotypical sequences in this task [7]. To evaluate whether neural representations of the stereotypical execution also existed in the SNr activity, we analyzed the activity of 731 neurons recorded while three highly trained animals performed motor sequences in the spatiotemporal task.

**FIGURE 2:**
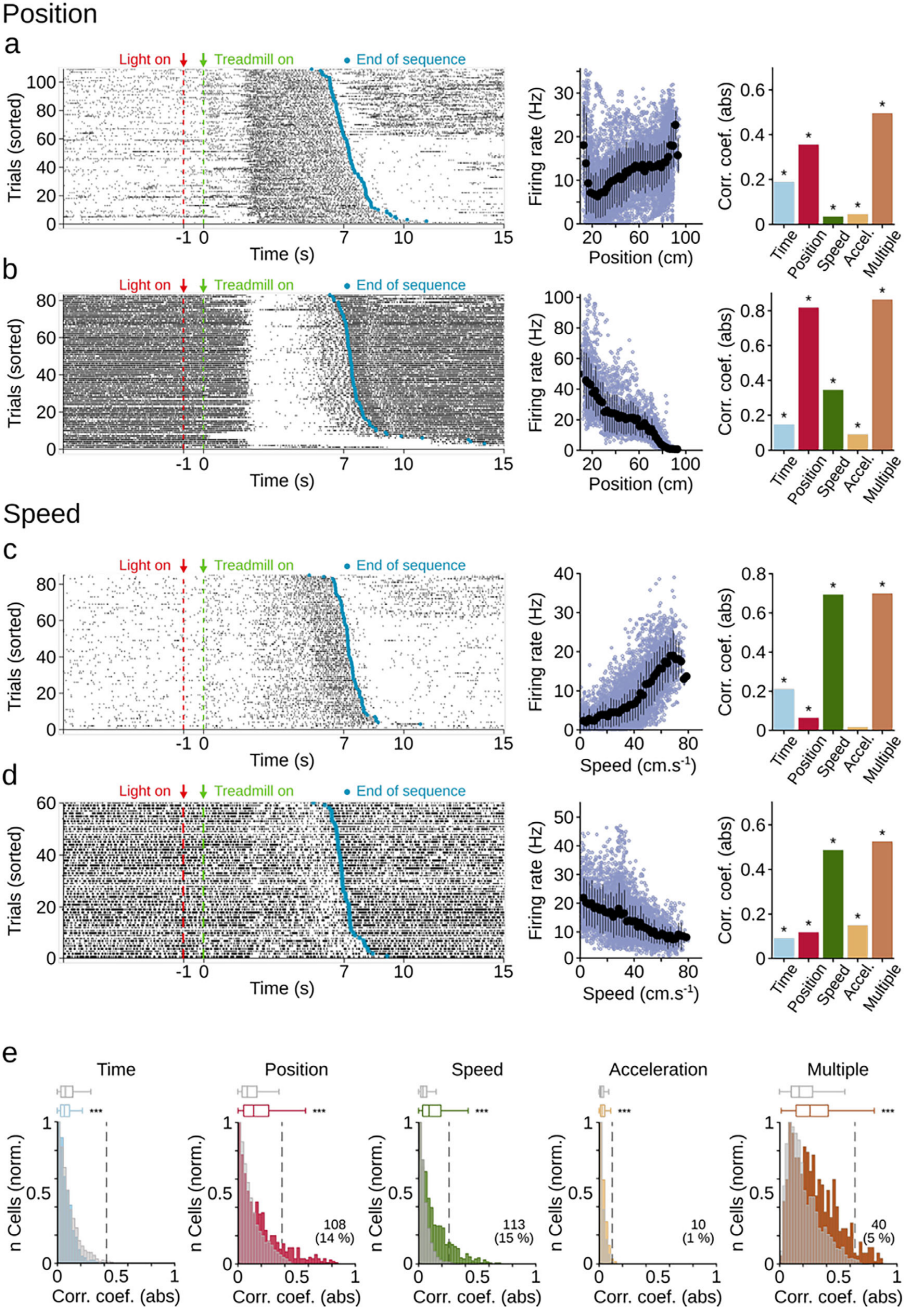
Linear and integrative representations of behavioral variables in the spatiotemporal task. (a–d) Four representative units with firing rates correlated with the position on the treadmill (a, b) or speed (c,d). Left, spike rasters aligned to the end of the motor sequence (a–d). Middle scatter plots show firing rate versus best correlated task variable (mean ± s.d.). Right, partial and multiple correlation coefficients between firing rate and task variables (*p < 0.001, Pearson's partial correlation coefficient). (e) Histograms displaying partial and multiple correlation coefficients (absolute values) between firing rate and task variables at the population level (same color code as a–e). Gray distributions represent partial and multiple correlation coefficients obtained with surrogated spike trains from the same neurons. The central line and box in the top boxplots (e) represent the median and 25th to 75th percentiles, and whiskers extend to the most extreme data points excluding outliers. *** *p* < 0.001 two‐sided Wilcoxon rank sum test.

First, we calculated Pearson's partial correlation coefficients between the firing rate of single cells and execution parameters of the motor sequence, such as position on the treadmill, running speed, acceleration, and time (Figure 2). As in the input BG, we found that a significant proportion of neurons presented linear correlations between SNr spiking activity and position. Interestingly, we observed different modulation patterns, with some neurons’ firing rates increasing (Figure 2a) and others decreasing (Figure 2b) depending on the rat's position on the treadmill (the specificity of these correlations was confirmed when plotting error trials; Figure S1). This might be related to the direct and indirect pathways being activated [15, 22]. Similarly, we found neurons whose higher partial correlation coefficient was related to running speed. Similar to position, we also found neurons with positive (Figure 2c) and negative (Figure 2d) linear correlations with running speed.

It has been shown that single neurons in the DLS multiplex execution variables, primarily locomotion speed and treadmill position [7, 18]. To evaluate the extent to which the spiking activity of SNr could also be explained by a linear combination of our behavioral variables, we applied multiple regression analysis. We found that individual neurons presenting high partial correlation values for speed and position also exhibited high multiple correlation coefficients (Figure 2a–d far right panels), suggesting that multiple execution variables influenced the activity of SNr neurons. We conducted an extended analysis using the Akaike index to estimate the information loss and compared regression models with all possible combinations of variability predictors [7]. This analysis revealed that, like the DLS, the animal's position and speed primarily explained the spiking activity in the SNr (Figure S2a). Next, to evaluate the prevalence of these execution representations in the general neuronal population of the SNr, we constructed surrogated spike trains by randomly adding ± 5 s to each spike of the original spike trains observed in each neuron. Then we performed the same partial and multiple regression analysis and repeated the operation 100 times, resulting in surrogated distributions for each regressor and the multiple regressor (Figure 2e, gray distributions). We statistically compared the observed and surrogated distributions at the population level and found that speed, position, and the multiple coefficients, but not time or acceleration, were significantly higher than the surrogated distributions (Figure 2e, color‐coded distributions). At the single‐cell level, we found that about 14%–15% and 5% of the neurons, respectively, expressed higher partial correlation and multiple regression values than the 99.5th percentile of the surrogated population (indicated by the dotted line).

**FIGURE 3:**
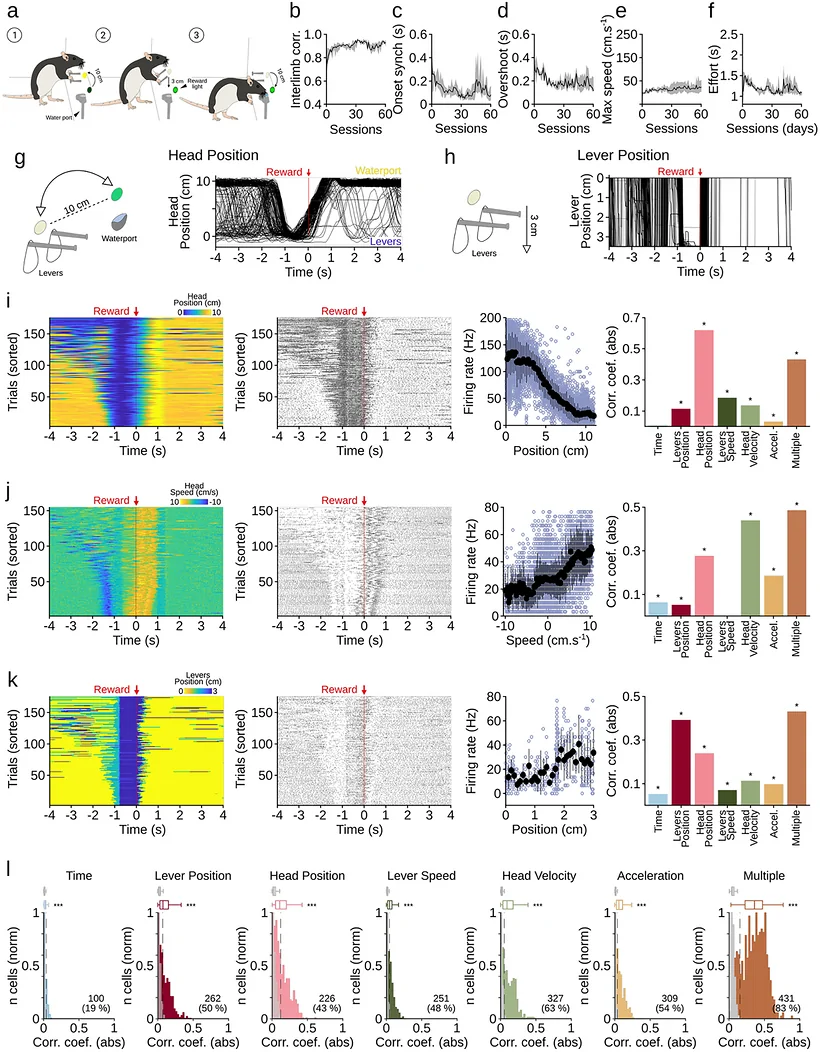
Linear and integrative representations of behavioral variables in the bimanual coordination task. (a) Schematic representation of the behavioral setup and protocol. Numbers indicate main phases of movement from levers to water port. (b–f) Learning curves in the bimanual coordination task for the following variables: interlimb correlations (b), onset synchrony (c), overshoots (d), maximum speed (e), and effort (f). Data are presented as median (solid line) + 75th and 25th percentiles (shaded area). (g) Schematic representation of general trajectory from levers to water port (left) and representative movement trajectories aligned to reward onset (red line and arrow). (h) Representative lever trajectories aligned to reward onset. (i) Trial‐by‐trial color‐coded instantaneous head/body positions (far left) and spike rasters (center left) aligned to reward onsets (red lines and arrows). Center right scatter plot shows firing rate versus task variable. Right end bar plot shows partial and multiple correlation coefficients between firing rate and task variables (*p < 0.001, Pearson's partial correlation coefficient). (j, k) Same as in h but for head/body velocity (I), or lever position (J). (l) Histograms displaying population partial and multiple correlation coefficients (absolute values) between firing rate and task variables (same color code as i–k). Gray distributions represent partial and multiple correlation coefficients obtained with surrogated spike trains from the same neurons. The central line and box in the top boxplots (I) represent the median and 25th and 75th percentiles, and whiskers extend to the most extreme data points excluding outliers. *** *p* < 0.001, two‐sided Wilcoxon rank sum test.

The previous results suggest that position and speed signals are continuously represented and preserved throughout the BG circuit, further supporting their implication in movement modulation and control [2, 7, 9, 18, 19]. In the current task, animals performed a sequence of locomotion accelerations and decelerations involving complex spatiotemporal adaptations and a sense of motor timing (Figure 1). If the BG were indeed implicated in the modulation and control of movement parameters, then we would expect to find these kinds of representations in another behavioral context with a different spatial and temporal content range. To test this hypothesis, after concluding the treadmill‐based task recordings, we trained the same rats in a second behavioral protocol, the bimanual coordination task [15, 18, 36, 37]. While in the first protocol, animals were required to perform locomotion runs constrained to a spatiotemporal range of hundreds of centimeters and dozens of seconds, in this second protocol, the same animals were required to perform short postural displacements (∼10 cm, right to left and left to right) and coordinated forelimb movements restricted to 3 cm in only hundreds of milliseconds. In this task, the spatial and temporal variables remained constant, but the execution of the movement required more coordination and dexterity in the forelimbs (Figure 3a). Briefly, rats were trained to simultaneously displace two levers vertically, one with each front paw. The rats had to simultaneously hold down both levers for at least 750 ms below a spatial threshold of 3 cm. Successfully coordinated movements were rewarded with a drop of water delivered through a water port located 10 cm to the right of the lever set. During bilateral task execution, animals were monitored with a video camera located above the setup, and the position of the levers was continuously recorded, allowing for continuous evaluation of multiple behavioral variables and reliable follow‐up throughout the learning curve. As previously reported [15, 18, 36, 37], training improved interlimb correlations (Figure 3b), defined as the correlation of the trajectories of left and right levers, a general indication of movement coordination and synchrony. Training also decreased bilateral movement onset synchrony (Figure 3c), an indicator of movement onset coordination. An efficient movement is also characterized by maintaining the levers below the spatial threshold only for the time needed to end the movements. Hence, to evaluate the duration of bilateral movements, we measured the time that the levers stayed below the spatial threshold after the reward was delivered (overshoot, Figure 3d). This variable also decreased with training. We also calculated the average maximum speed of movement trajectories for each session, a variable that remained stable throughout training (Figure 3e). Finally, we estimated the effort to obtain a single reward, which we defined as the amount of time each lever was pressed to obtain one drop of water (Figure 3f). Animals also expressed stereotypical movement transitions between the water port and the lever set (Figure 3g) and stereotypical lever trajectories reflecting coordinated forelimb movements (Figure 3h). Because we wanted to record the same animals in both tasks, we started SNr recordings in the bilateral coordination task only 5–10 sessions after the beginning of training; that is, animals in this task were not fully trained but were already performing more than 100 trials per session. Keeping this caveat in mind, we aimed to determine whether the kinematic representations remain intact during movement execution within this behavioral context. Like in the spatiotemporal task, we calculated Pearson's partial correlation coefficients between the firing rate of individual cells and each behavioral variable, including time, lever position, lever speed, head position, head velocity, and head acceleration. As in the previous case, we also estimated the possibility that multiple variables were responsible for the variability in the spiking activity by applying multiple regression analysis. In line with our spatiotemporal task findings, the variables associated with position and speed of movement of both the head and the levers were strongly represented in the SNr's neuronal activity (Figure 3i–k). We also performed multiple regression analysis to evaluate whether a linear combination of the main behavioral variables could explain the firing rates of SNr neurons. Interestingly, we found that neurons also displayed higher partial correlation coefficients for position and speed (for both general head displacement and lever pressing) than the surrogated distributions, which is similar to what we observed in the spatiotemporal task (Figure 3l). The same phenomenon occurred for the integration of multiple variables. In this case, however, upon applying the Akaike index to estimate information loss and comparing regression models with different combinations of variability predictors, we discovered that lever and head position combinations were the most robust variables explaining spiking variability (Figure S2b).

**FIGURE 4:**
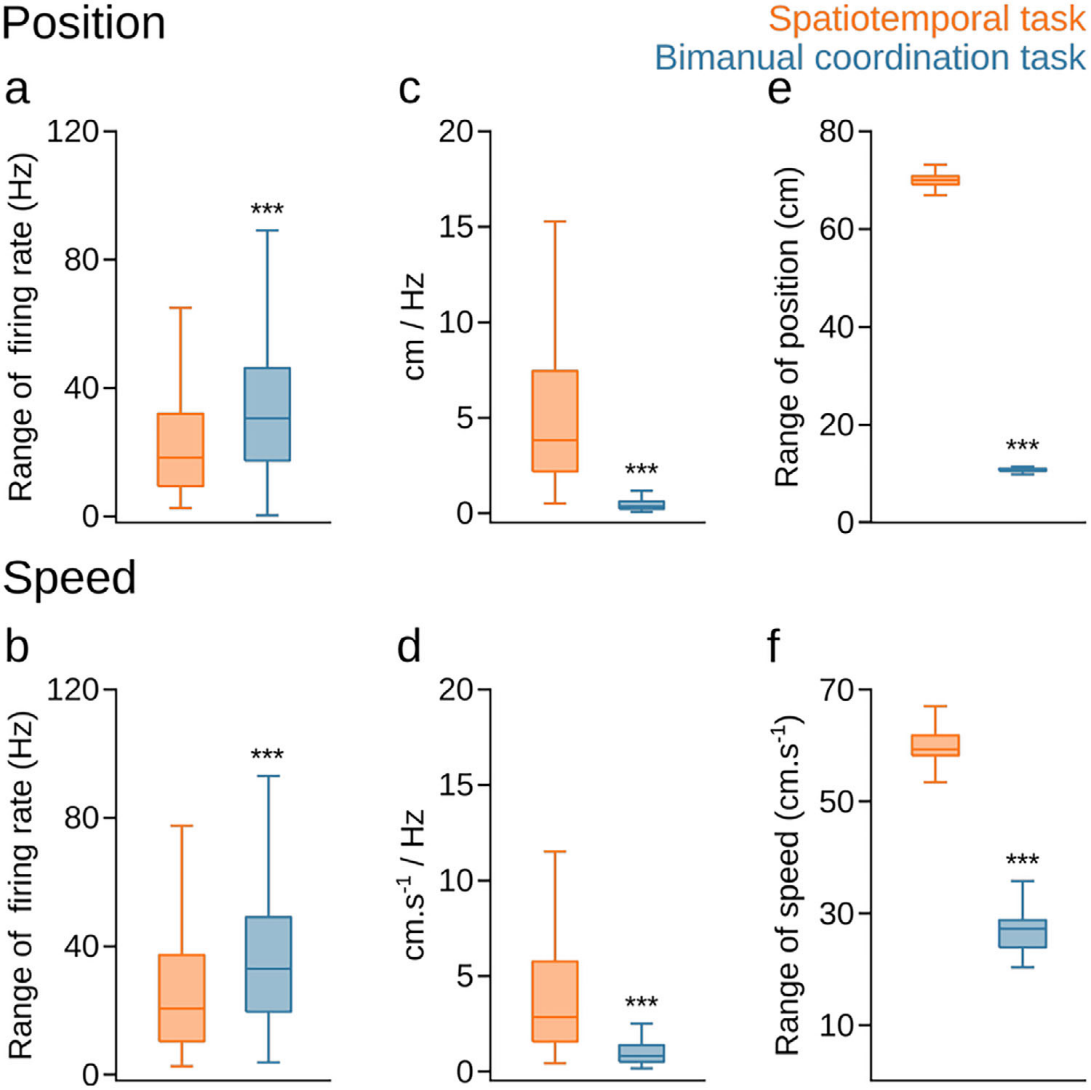
Scaling code in the SNr. Firing rate range in the spatiotemporal and bimanual coordination tasks (color coded) for neurons classified as position‐ (a) or speed‐related (b). Same as in a, b, but for position (c) or speed (d) content by unit of frequency. Range of position (e) or speed (f) expressed during behavioral sessions. Data correspond to neurons that expressed significant partial correlation coefficients (*p* < 0.001) with speed or position in either task. The central line and box in boxplots represent the median and 25th and 75th percentiles, and whiskers extend to the most extreme data points excluding outliers. *** *p* < 0.001, two‐sided Wilcoxon rank sum test.

Our experiments indicate that the animal's movement position and speed in both tasks are robustly encoded in the activity of SNr neurons. However, how does SNr spiking activity adjust to these markedly different behavioral contexts? One possibility could be that SNr activity range differs in both behavioral contexts, such as lower firing rates in the task with less spatial content. Another possibility is that the SNr displays similar activity ranges independently of the spatiotemporal characteristics of the behavioral protocol. This second possibility supports the notion of a context‐dependent, scalable representation of these variables, rather than a rigid or fixed relationship between the firing rate, articulation angles, and torques. We used our SNr recordings of the same animals during the behavioral execution in the two different protocols and calculated the activity range between the minimum and maximum firing rates of units that expressed significant partial correlation coefficients for position or speed during the execution of the movement sequence in one or both tasks. First, we found that SNr neurons classified as position‐ or speed‐selective presented higher firing rate ranges in the bimanual coordination task than in the spatiotemporal task (Figure 4a,b). Then, we calculated the firing rate change for each unit of position or speed. We found that, for the “position cells,” each unit of rate activity (Hz) encoded an average of about 3.8 cm of space in the treadmill. In the bimanual coordination task, each Hz encoded about 0.35 cm of space (Figure 4c). On the other hand, each unit of rate encoded about 2.84 and 0.81 cm s^−1^ of speed in the treadmill and bimanual task, respectively (Figure 4d). This means that between tasks, the SNr scaled their range about 10.91 times for position and 3.51 times for speed. It is worth noting that when we calculated the range of position and speed displayed during the same recording sessions, we found that the difference between tasks was 6.44 times for position and 2.17 times for speed (Figure 4e,f). This indicates a nonlinear scaling of SNr neuronal activity depending on the behavioral context. When transiting to a task where spatial content was smaller, position‐ and speed‐classified units increased their firing rate about 1.6 times, suggesting a compensation to more accurately encode both variables. Then, we repeated the same analysis in the neurons with the highest partial correlation values for position and speed (Figure S3). These were defined as units with values above the 99.5th percentile of surrogated distributions (depicted in Figure 2e and Figure 3l; treadmill 108 and 113 neurons; bimanual coordination task, 226 and 327, for position and speed, respectively). This subpopulation presented similar values, except that for speed, their scaling matched the actual change in behavioral space almost perfectly, with a rescaling of 2.27 times for the speed‐related firing rate and 2.16 times for the actual change in the range of speed (Figure S3).

**FIGURE 5:**
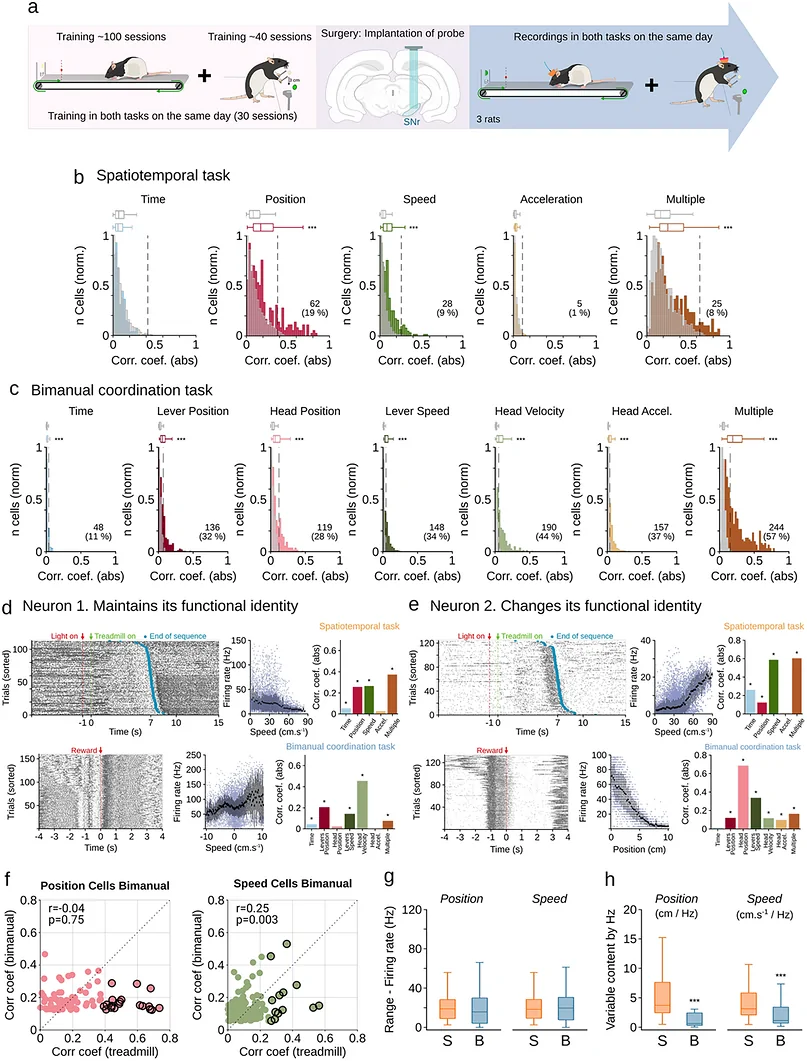
Execution variables encoded in the same neurons in both protocols. (a) Schematic representation of training and recording sessions to record neural activity from the same neurons in both tasks. Histograms displaying population partial and multiple correlation coefficients (absolute values) between firing rate and task variables during treadmill (b) and bimanual task (c) execution. Gray distributions represent partial and multiple correlation coefficients obtained with surrogated spike trains from the same neurons. (d) A representative unit with the same functional identity in both tasks is presented. Spike rasters aligned to the end of the motor sequence in the bimanual coordination task (top row) or reward onset in the bimanual coordination task (bottom row). Middle scatter plots show firing rate versus best correlated task variable (mean ± s.d.). Right, partial and multiple correlation coefficients between firing rate and task variables (*p < 0.001, Pearson's partial correlation coefficient). (e) Same as in d, but for a unit that changed its functional identity between tasks. (f) Scatter Pearson correlation plots between partial correlation coefficients obtained during the execution in both tasks for position‐classified (left) or speed‐classified cells (right) (classified during the bimanual coordination task). (g) Firing rate range in the spatiotemporal and bimanual coordination tasks (color‐coded) for position‐ or speed‐classified cells. (h) Position or speed content by unit of firing frequency for units classified as position‐ or speed‐related. The central line and box in boxplots (b, c, g, h represent the median and 25th and 75th percentiles, and whiskers extend to the most extreme data points excluding outliers. ***p < 0.001, two‐sided Wilcoxon rank sum test.

Our findings indicate that within the BG, signals related to position and speed are maintained in both tasks, yet their modulation varies depending on the context. This suggests that these signals are involved in an abstract representation of movement control, more than the control of specific torques and angles of articulations or joints. However, these data pose new questions, such as whether a single neuron encodes the same parameter (speed or position) for both tasks or if SNr signals are segregated by their output targets. For example, does the SNr encode a specific parameter projecting to the motor thalamus or to other important SNr targets? While the same animals were recorded in both tasks, we collected the data during different sessions and phases of the learning curve. This made it difficult for us to determine whether the same neurons were encoding the same or different variables in both tasks, i.e., whether they were maintaining or changing their functional identity, or if rescaling between behavioral contexts occurred at the individual or population level. To address this issue, we trained a new group of four animals in both tasks, in a way that each animal would perform one task after the other in the same day and session, increasing the chance to record the same neurons in both conditions (Figure 5a). Briefly, animals were trained only in the treadmill task until they entered the variable speed phase of the protocol. At that point, we restricted the animals' access to water and initiated daily training in both the bimanual coordination task and the treadmill task. Animals were always subjected to the bimanual task first, and immediately after the session (about 45 min, 150–200 trials), they were transported to the treadmill setup for a regular session in this protocol. We started electrophysiological recordings after at least 30 sessions in each task, and we monitored SNr activity daily in both tasks. We were able to record 432 neurons in these animals, of which 323 were stable in both tasks. Similar to the previous group, individual SNr neurons recorded during treadmill execution encoded mainly position and speed in both tasks (Figure 5b,c). Then, we explored whether the same neurons encoded the same or different variables in both tasks. We found units that maintained their functional identity independently of the task‐that is, encoded the same behavioral variable in both tasks (Figure 5d). However, we also found instances where neurons encoded distinct variables based on the task, such as speed on the treadmill and position in the bimanual task (Figure 5e). Additionally, we observed neurons that encoded behavioral variables for a single task. In general, 80% of neurons encoded a behavioral variable at least in one task, while only 20% showed no significant modulation in either protocol. Importantly, from the neurons that encoded at least one variable, 14% maintained their functional identity, and 18% changed it. We confirmed these data by calculating Pearson correlation coefficients between the partial correlation values obtained in both tasks, finding no clear relationship for position‐classified units and a discrete but significant relationship for speed‐modulated units (Figure 5f). Finally, we calculated the firing rate range for each unit of position or speed and found similar results to those obtained in the original group (Figure S3), with an approximately eight‐fold re‐escalation for position and an approximately three‐fold re‐escalation for speed (Figure 5g,h).

**FIGURE 6:**
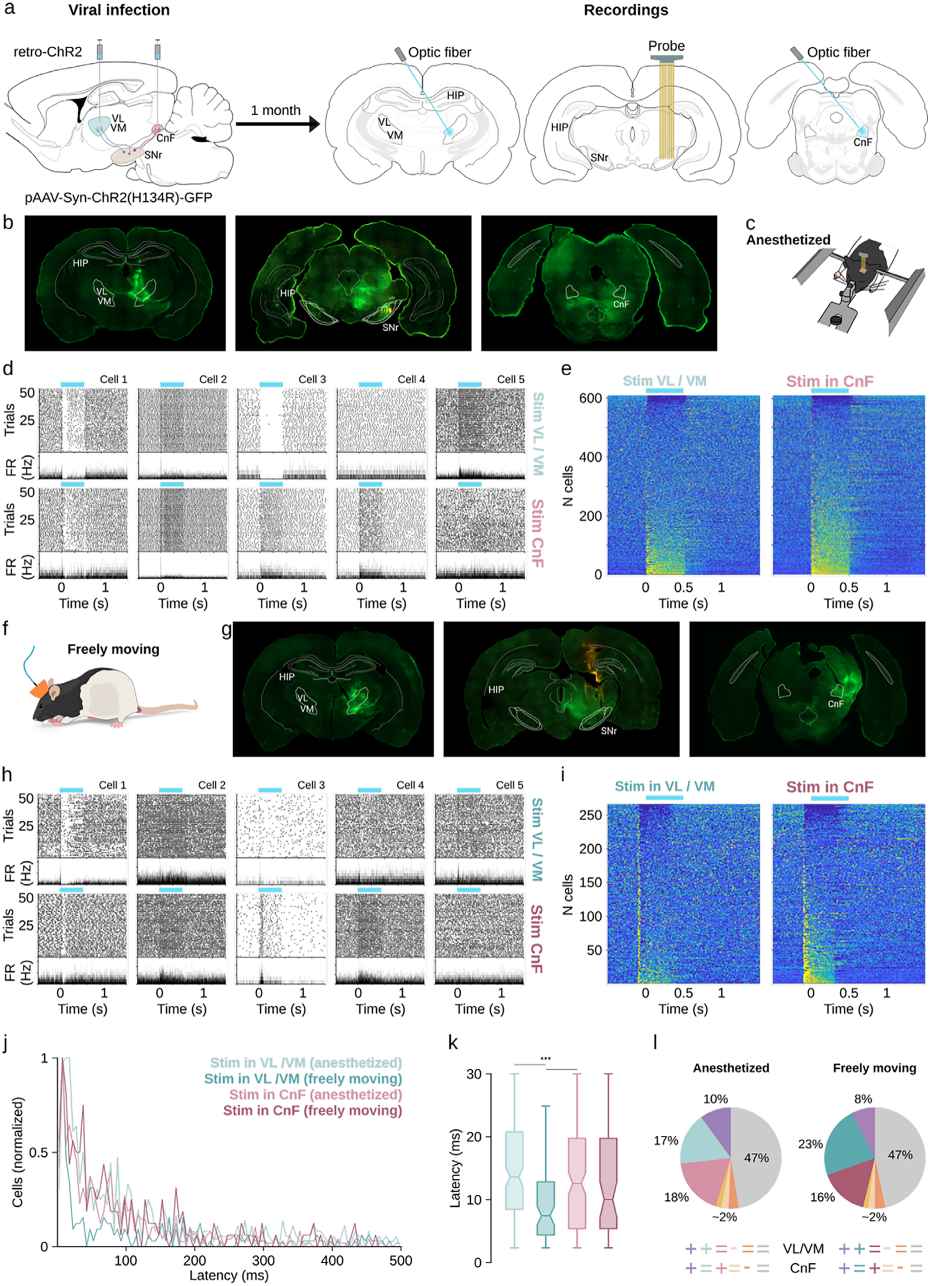
Targeting SNr neurons projecting to the VL/VM and CnF. (a) Schematic representation of the infection and recording strategy, optical fibers (angled), and microelectrode implantation in the SNr. (b) Histological confirmation of infection (Green fluorescent protein, GFP) and recording sites (DiI stained, red channel) in anesthetized animals (c). HIP, hippocampus. d) Representative SNr spike rasters (top) and average perievent histograms (bottom) for electrophysiological recordings and optical manipulations in anesthetized rats. Activity in the raster plots is aligned to the onset of optical stimulation (500 ms stimulus indicated by blue bar) delivered into the VL/VM (upper row) or the CnF (lower row) ipsilateral to the recording site. (e) Averaged (z‐scored, color‐coded) firing rates evoked by ipsilateral VL/VM (left) or CnF (right) stimulation, for cells recorded in the SNr of anesthetized animals. Activity is sorted according to the maximum averaged firing rate reached during the 500 ms of stimulation (blue bars). (f–i) Same as in b–e but for freely moving rats. (j) VL/VM or CnF evoked response latencies in anesthetized or freely moving animals (color coded). (k) Comparison of the response latencies. The central line and box in boxplots represent the median and 25th–75th percentiles, and whiskers extend to the most extreme data points excluding outliers (Bonferroni post hoc test, *p* < 0.05). (l) Pie plots representing the percentage of SNr cells with short‐latency responses to the VL/VM, CnF, or both, or neurons that decreased their activity with stimulation (color coded).

The previous data suggest that SNr neurons encode specific variables depending on the behavioral context. Our tasks differ not only in the spatiotemporal content but also in the action required to obtain a reward. On the one hand, the treadmill‐based protocol allows the animals to control the timing and speed of locomotion, while in the second task, skillful and coordinated forelimb movements are required. Previous studies have proposed that BG control of locomotion may be achieved through direct SNr projections to the locomotor mesencephalic regions [14, 38, 39] without thalamic relays. On the other hand, Hikosaka [12] proposes that behaviors involving skillful forelimb movements may implicate SNr projections to the motor thalamus, thereby impacting motor cortical regions. These antecedents suggest that different SNr neuron subpopulations or projections may relate to SNr behavioral control in our two protocols. To address this possibility, we implemented an optogenetic‐based method to identify SNr neurons projecting to the motor thalamus (VL/VM) and cuneiform nucleus (CnF) of the mesencephalic locomotor region in anesthetized animals. In brief, we expressed channelrhodopsin 2 (ChR2 and green fluorescent protein GFP as a fluorescent reporter) in SNr neurons projecting to both the VL/VM and CnF by injecting the retrograde virus pAAV‐Syn‐ChR2(H134R)‐GFP in both target regions (Figure 6a). Four weeks after injections, we observed GFP‐positive signals in the SNr, VL/VM, and CnF (Figure 6b). Then, during SNr electrophysiological recordings, we induced antidromic responses by performing optical stimulation (500 ms stimulus) of the VL/VM or CnF to determine if SNr neurons projected to one or both nuclei and find out if SNr neurons projected to one or both nuclei (Figure 6a,c). We were able to record the activity of 609 neurons from five anesthetized animals. Stimulation in both structures induced different SNr responses visible at the individual (Figure 6d) and population levels (Figure 6e), with neurons robustly increasing and decreasing their firing rates during the 500 ms stimulation periods. Next, we implemented the same procedure in freely moving animals that had been trained in our behavioral protocols. We were able to record 266 neurons from three animals and found broadly similar results (Figure 6f–i). We also calculated the response latencies to both stimulation sites (VL/VM and CnF) and experimental conditions (anesthetized and freely moving) (Figure 6j). At both stimulation sites, we found neurons with latencies shorter than 15 ms, suggesting direct projection to the stimulated region. However, at the population level, the shortest latencies were found when stimulating the VL/VM (Figure 6k). Then, we quantified the neurons that presented short latencies to the VL/VM, the CnF, or both. About 8%–10% of the population responded to the stimulation of both sites, and 16% to 23% of the neurons responded to stimulation at only one nucleus. We observed minor proportions of neurons inhibiting their activity with either combination (Figure 6l). Altogether, these data suggest that SNr neuron subpopulations project to the VL/VM, CnF, or both. They also suggest that lateral inhibition in the SNr may play a minor role in shaping the output of this nucleus. Finally, previous reports have shown that, by analyzing the spike wave shapes of neurons in the SNr, it is possible, in principle, to approximate their lineage identity, that is, principal GABAergic projecting neurons (pSNrGNs) vs. dopaminergic neurons (pSNrDNs) [25, 40]. Hence, to evaluate the possibility that these types of neurons would be associated with a particular target region, that is, VL/VM or CnF, we applied a PCA‐based classification method (see Section 4) [18, 40, 41, 42] to the average spike shapes of neurons recorded under freely moving conditions. Spike wave shapes were best classified into two clusters (Figure S4a,b). Consistent with previous reports, the largest cluster contained almost 80% of the recorded neurons and presented sharp spikes. Hence, this cluster was considered to represent pSNrGNs, and the second cluster was considered to represent pSNrDNs (Figure S4c,d) [18, 40, 41, 42]. We observed that pSNrGNs expressed similar proportions of putative projecting neurons to VL/VM or CnF (Figure S4e) as the general population (Figure 6l). However, most units classified as pSNrDNs (94%) presented no significant responses to light stimulation of either nucleus (Figure S4f), suggesting that this population was composed of nigrostriatal neurons, mainly projecting to BG nuclei.

**FIGURE 7:**
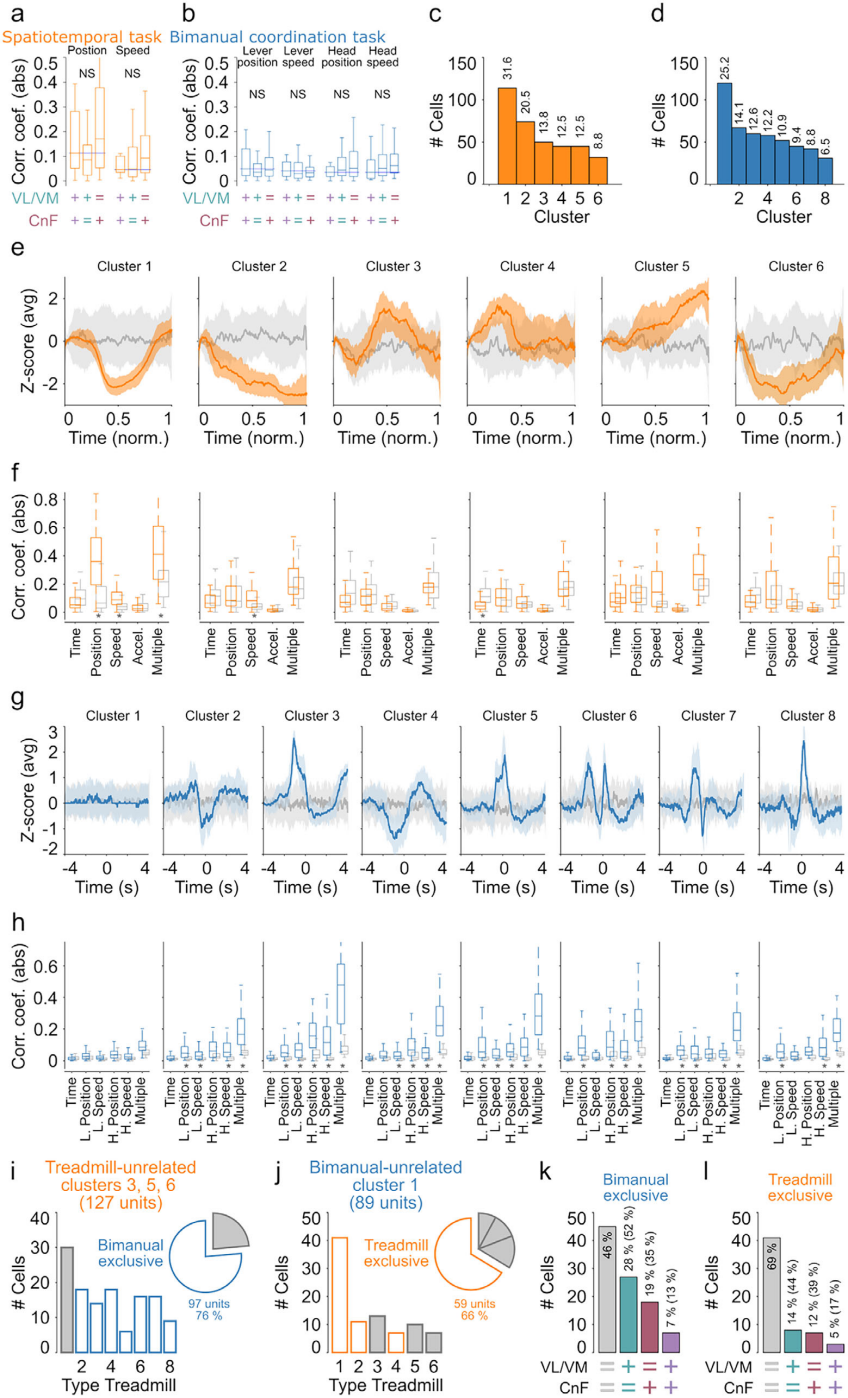
SNr neurons have similar encoding properties independently of their target structure. Partial correlation coefficients for neurons responding to VL/VM, CnF, or both, VL/VM + CnF, stimulation during behavioral execution in the spatiotemporal task (a) and the bimanual coordination task (b). Number (bars) and percentage (indicated on each bar) of SNr neurons classified into each cluster based on the activity patterns expressed during behavioral execution in the treadmill‐based spatiotemporal protocol (c; orange color scheme throughout the rest of the figure) or the bimanual coordination task (d; blue color scheme throughout the rest of the figure). (e) Average behaviorally evoked peri‐event histograms for neurons belonging to each cluster during treadmill execution. (f) Partial and multiple correlation coefficients (absolute values) for neurons classified into each cluster during treadmill execution. (g, h) Same as in (e, f), but for activity recorded during behavioral execution in the bimanual coordination task. Colored and gray traces (g and e) and box plots (f and h) represent observed and surrogate activity from the same neurons, respectively. Box plots indicate the median and the 25th and 75th percentiles. Significant differences between observed and surrogate data are indicated by asterisks below the corresponding box plots (Bonferroni post hoc test, *p* < 0.05). (i) Distribution of bimanual coordination response patterns for neurons classified as unrelated to treadmill execution (indicated at the top). The pie chart shows the number (and percentage) of neurons tuned (blue) to bimanual coordination execution. (j) Distribution of treadmill‐related response patterns for neurons classified as unrelated to bimanual coordination execution (indicated at the top). The pie chart shows the number (and percentage) of neurons tuned (orange) to treadmill execution. Number (bars) and percentage (indicated on each bar) of SNr neurons exhibiting short‐latency responses to stimulation of axon terminals in VL/VM, CnF, or both (color coded as in Figure 6l) for neurons exclusively tuned to bimanual (k) or treadmill (l) execution. Percentages in parentheses indicate the proportions excluding neurons unresponsive to stimulation of either target structure.

After determining that SNr neurons may be segregated into subpopulations by their outputs to the VL/VM and CnF, we investigated whether these populations preferentially represented different parameters of movement in our two behavioral contexts. First, we calculated partial correlation coefficients in both tasks, and consistent with our previous data, speed and position were the variables with the highest partial correlation values for these neurons (Figure S4). Then, we grouped neurons based on whether they responded to stimulation in both target regions (VL/VM+CnF) or just one region (VL/VM or CnF) and performed inter‐subgroup comparisons. We found that speed and position partial correlation values did not show significant differences for any of the neuron subgroups during behavioral execution on the treadmill (Figure 7a; position: K–W, *df* = *2, X^2^* = *5.91, p* = *0.52;* speed: K–W, *df* = *2, X^2^* = *3.12, p* = *0.209;*) or in the bimanual coordination task (Figure 7b; *lever position*: K–W, *df* = *2, X^2^* = *0.44, p* = *0.802; lever speed*: K–W, *df* = *2, X^2^* = *1.32, p* = *0.51; head position*: K–W, *df* = *2, X^2^* = *1.46, p* = *0.48; head speed*: K–W, *df* = *2, X^2^* = *4.99, p* = *0.08;*). Similar results were obtained for neurons classified as pSNrGNs or pSNrDNs (Figure S4g,h). These results encompass all neurons recorded in both protocols and assume linear relationships with different execution variables. However, there's still the possibility that neurons in the SNr may be segregated into functional subpopulations, not necessarily expressing linear relationships with a particular variable, but for example, tuned to a particular behavioral protocol. To address this possibility, we first applied a classification method based on Euclidean distances between clusters derived from the principal components of the average response patterns of each neuron across both behavioral protocols [15, 18, 41] (see methods). Using this approach, we found that the same SNr neurons could be classified into six response patterns (clusters) during execution of the spatiotemporal task (Figure 7c) and into eight response patterns during execution of the bimanual coordination task (Figure 7d). To determine whether these response patterns were functionally linked to the execution of each protocol, we calculated the partial correlation coefficients for each cluster and compared them with the corresponding partial correlation coefficients obtained from surrogate spiking activity generated from the same neurons, preserving the number of spikes. In the spatiotemporal protocol, clusters 1, 2, and 4 exhibited significantly higher partial correlation values than the surrogate activity for at least one behavioral variable (Figure 7e,f. Cluster 1: K–W, *df* = *9, X^2^* = *613.69, p* < *0.001;* Cluster 2: K–W, *df* = *9, X^2^* = *345.23, p* < *0.001;* Cluster 4: K–W, *df* = *9, X^2^* = *215.26, p* < 0.001), and together, these three clusters accounted for more than 64% of the population. In contrast, clusters 3, 5, and 6 did not differ significantly from surrogate activity for any of the behavioral variables (Figure 7e,f. Cluster 3: K–W, *df* = *9, X^2^* = *276.20, p* < *0.001;* Cluster 5: K–W, *df* = *9, X^2^* = *209.94, p* < *0.001;* Cluster 6: K–W, *df* = *9, X^2^* = *134.06, p* < 0.001). The same analysis revealed that during execution of the bimanual coordination task, neurons belonging to clusters 2–8 (accounting for more than 70% of the population) exhibited significant differences relative to surrogate activity for at least one behavioral variable, whereas neurons belonging to cluster 1 did not (Figure 7g,h. Cluster 1: K–W, *df* = *11, X^2^* = *525.78, p* < *0.001;* Cluster 1: K–W, *df* = *11, X^2^* = *525.78, p* < *0.001;* Cluster 2: K–W, *df* = *11, X^2^* = *357.46, p* < *0.0001;* Cluster 3: K–W, *df* = *11, X^2^* = *396.87, p* < *0.0001;* Cluster 4: K–W, *df* = *11, X^2^* = *312.68, p* < *0.001;* Cluster 5: K–W, *df* = *11, X^2^* = *314.62, p* < *0.001;* Cluster 6: K–W, *df* = *11, X^2^* = *261.61, p* < *0.001;* Cluster 7: K–W, *df* = *11, X^2^* = *237.16, p* < *0.001;* Cluster 8: K–W, *df* = *11, X^2^* = *197.14, p* < 0.001).

These findings suggest that neurons composing clusters 3, 5, and 6 during treadmill execution and cluster 1 during bimanual execution did not functionally participate in the corresponding behavioral protocol. We therefore evaluated whether neurons belonging to the treadmill‐unrelated clusters (3, 5, and 6) were functionally linked to any behavioral variables during execution of the bimanual task. We found that 76% of the treadmill‐unrelated units (97 neurons) belonged to functional bimanual clusters (Figure 7i), indicating that these neurons were selectively tuned to behavioral variables in the bimanual coordination protocol. Then, the reciprocal analysis for neurons belonging to the bimanual‐unrelated cluster 1 revealed that most of these units (66%, corresponding to 59 neurons) belonged to the functional treadmill clusters 1, 2, and 4 (Figure 7j), indicating that these neurons were selectively tuned to behavioral variables in the spatiotemporal protocol. Then, we investigated whether neurons exclusively tuned to one protocol exhibited a preference for a particular projection target. We found that 46% of treadmill‐exclusive neurons and 69% of bimanual‐exclusive neurons were not optogenetically identified. Several technical factors could account for this, for example, the distance between axonal terminals and the light source. Among the remaining units, we found no robust relationship between neuronal type and thalamic or mesencephalic projection targets (Figure 7k,l).

The previous results confirmed that in both protocols speed and position are robustly encoded by a significant amount of SNr neurons, which opens the question of whether, as a population, SNr activity may also reflect these variables. To address this question, estimated the extent to which different execution variables were represented in SNr population dynamics across both tasks. For this, we analyzed the activity of the neurons that were recorded during both tasks and performed a modified version of the analysis reported by Taouali et al. [43]. For the treadmill condition, we first selected trials with durations close to the target time of 7 s, specifically those ranging from 6 to 9 s, which represented more than 90% of all successful trials. We then normalized the duration of the running periods and extracted, for each neuron, the average spiking activity pattern across trials. We applied the same procedure to the bimanual coordination task, except that time normalization was unnecessary because successful trials in this protocol required a fixed holding period of 750 ms (Figure S6a,b). Once the spiking activity patterns had been extracted for each neuron, we performed principal component analysis (PCA) on the activity of all neurons and evaluated how the resulting population dynamics evolved over the course of the trial with respect to the main behavioral variables. These variables included position, speed, and acceleration in the treadmill task (Figure S6a), and lever position, head position, and movement speed in the bimanual coordination task (Figure S6b). To capture population variability rather than relying on a single population estimate, we performed 100 iterations in which, for each iteration, 50 neurons were randomly selected from the entire pool of recorded neurons. We report both the population dynamics of the complete dataset and those obtained from the 100 subsampled populations of 50 neurons.

A first notable difference between tasks was that, in the treadmill protocol, the first three principal components accounted for more than 90% of the population variance (Figure S6c), whereas in the bimanual coordination task the first three principal components explained approximately 30% of the variance (Figure S6d). We then evaluated whether these three principal components were correlated with the corresponding execution variables. For each of the 100 iterations, we extracted the average trajectories of the behavioral variables from the sessions contributing the randomly selected neurons. For the full‐population analysis, we calculated the average behavioral trajectories across all sessions. Overall, Pearson correlation values between the principal components and behavioral variables were high in both protocols. In the treadmill task, PC1 exhibited the strongest correlations with running speed and, to a lesser extent, with the animal's position (Figure S6e). In contrast, during the bimanual coordination task, the strongest correlations with PC1 were observed for head position and, subsequently, lever position (Figure S6f). PC2 in the treadmill task showed the highest correlation with position (Figure S6g), whereas in the bimanual task it correlated most strongly with movement speed (Figure S6h). Finally, PC3 in the treadmill task exhibited the strongest correlations with speed and acceleration (Figure S6i), whereas in the bimanual task correlation values were low and did not differ significantly among behavioral variables (Figure S6j). These analyses confirm that speed and position are strongly represented not only at the level of individual neurons but also in SNr population dynamics. Importantly, however, the population dynamics differed between tasks in two main respects. First, PCs 1–3 accounted for substantially more population variance during treadmill execution than during bimanual coordination. Second, PC1 in the treadmill task was more strongly correlated with locomotor speed, whereas in the bimanual coordination task it was more strongly associated with positional variables. This distinction is particularly relevant because PC1 accounted for the largest proportion of population variance in both tasks.

**FIGURE 8:**
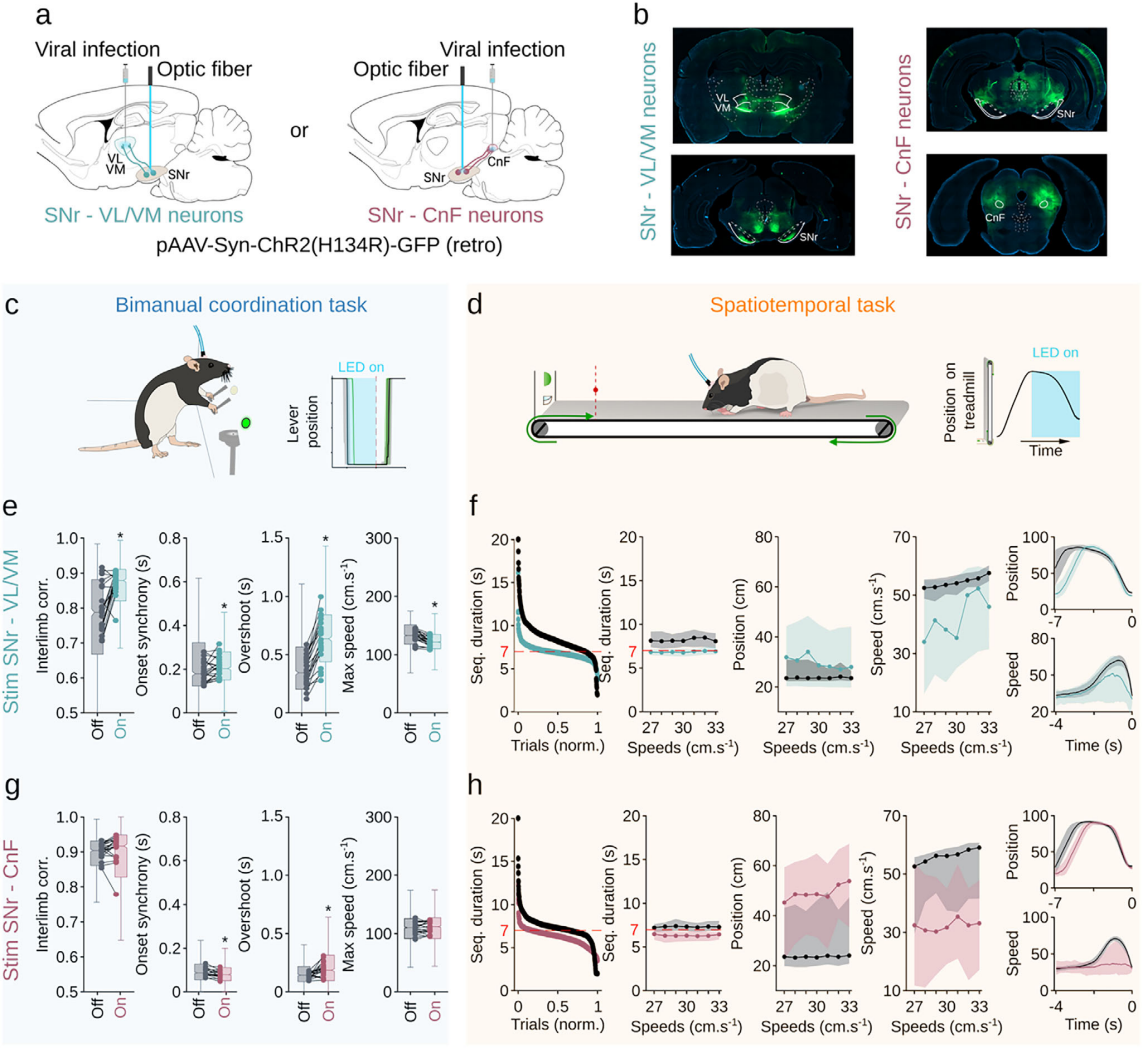
Targeting SNr neurons projecting to the VL/VM and CnF during behavioral execution. (a) Schematic representation of the infection and stimulation strategy. (b) Histological confirmation of infection sites (Green fluorescent protein, GFP) in the VL/VM (left) and CnF (right). Schematic representation of the moment of stimulation delivery during behavioral execution in the bimanual coordination task (c) and the spatiotemporal task (d). (e) From left to right, for a representative animal injected in VL/VM, interlimb correlations, onset synchrony, overshoot, and maximum speed for non‐stimulated (black code for the rest of the figure) and stimulated trials (colored code, for the rest of the figure). Box plots indicate median and 25th and 75th percentiles for all trials from all stimulated sessions; pairs of dots united by lines indicate medians of each session. (f) For the same animal as in e, from left to right behavioral variables for stimulated and non‐stimulated trials during execution in the spatiotemporal protocol: sorted sequence durations for all non‐stimulated and stimulated trials; median (solid lines) and 25th and 75th percentiles (dashed areas) for sequence duration, position and maximum speed displayed at different treadmill speeds; average postion (top) and speed (bottom) trajectories for all stimulated and non‐stimulated trials. (g, h) Same as in e and f, but for a representative animal injected in CnF.

The previous results confirmed that in both protocols speed and position are the primary variables encoded by SNr neurons and suggest that the abstract representations of these variables are broadcast throughout parallel but independent channels to downstream regions, depending on behavioral context. To evaluate if these parallel pathways would differentially influence behavioral control, we trained two new groups of animals on both tasks. After extensive overtraining, animals received injections of the virus pAAV‐SynChR2(H134R)‐GFP into either the VL/VM (*n* = 2) or the CnF (*n* = 3), enabling the retrograde expression of ChR2 in SNr projection neurons. Optical fibers were then implanted targeting the SNr (Figure 8a,b). Following an incubation period of more than 30 days, animals underwent experimental training sessions in which SNr optic stimulation was applied in 50% of randomly selected trials during behavioral execution in both tasks. In the bimanual coordination protocol, optical stimulation was triggered by a minimal lever displacement (> 0.1 mm), maintained while either lever was pressed, and terminated upon reward delivery (Figure 8c). In the spatiotemporal protocol, continuous optical stimulation was delivered during holding and acceleration phases of the movement sequence (Figure 8d; phases 2 and 3 in Figure 1a). Optical stimulation of SNr neurons projecting to the VL/VM produced clear behavioral effects in both protocols (Figure 8e,f). In the bimanual coordination task, stimulated trials showed greater bilateral synchrony, larger overshoots, and reduced movement speed (Figure 8e). In contrast, the same manipulation in the spatiotemporal task led to shorter movement sequences, reduced holding times (phase 2), and marked decreases in speed during the final phase of movement (Figure 8f). Conversely, optical stimulation of SNr neurons projecting to the CnF produced similar effects across both protocols (Figure 8g,h), though these effects were less pronounced in the bimanual coordination task (Figure 8g). Across all animals, stimulation of SNr projection neurons to either nucleus consistently induced robust behavioral changes in all variables of the spatiotemporal protocol (Figure S7a). However, behavioral effects in the bimanual coordination task were less consistent across subjects (Figure S7b). These results suggest that parallel SNr signals exert similar effects across behavioral contexts, mainly impacting speed control.

## 3 Discussion

Growing evidence shows that the BG encodes moment‐to‐moment representations of ongoing movements, such as speed, position, or amplitude [7, 15, 18, 19, 25, 26, 43, 44]. This suggests that its role in movement goes beyond selecting and initiating signals [4, 16, 17]. In this context, the striatum, which is the main input of the BG, hosts a variety of signals, including sensory‐related signals [9, 45, 46, 47], start and stop signals [4, 16], time‐related signals [8, 9, 48], vigor, speed, and amplitude signals [7, 19, 44], and reward‐related signals [49]. Researchers estimate that the rat's dorsal striatum, which integrates inputs from multiple and vast cortical and thalamic areas, contains about 2.7 million neurons, while the main output nucleus of the BG, the SNr, contains about 26 000 neurons [35]. This organization suggests a funneling function where the SNr integrates the previously mentioned heterogeneous signals into a common output signal. In this work, we show that SNr neurons linearly encode behavioral variables, mainly the animal's speed and position, for two behavioral protocols with different spatiotemporal contents, suggesting that the striatum integrates these representations. Recordings of the same animals and neurons in both behavioral protocols revealed that these representations scaled between behavioral contexts. This indicates that the SNr may represent these parameters as abstract entities applicable to different effectors. We adopted the conceptual term “abstract” from the classical motor control literature [50, 51, 52], where it has been linked to the concept of “motor equivalence,” generally defined as the central nervous system's capacity to achieve a “movement goal” using different muscles, joint angles, or body effectors. This concept implies that, hypothetically, actions are planned independently of the final effector, that is, as abstract entities. In this context, the fact that SNr activity scales representations of speed and position according to the spatiotemporal context indicates that these parameters are not represented in a manner that is fixed to a particular set of body effectors. Instead, it suggests that not only the action itself, but also its kinematic parameters, are represented at an abstract level. We also explored whether SNr‐projecting neurons could be organized into subpopulations that target distinct thalamic and mesencephalic motor regions. By combining pathway‐specific optogenetic manipulation with multiunitary recordings, we found groups of neurons that projected to the VL/VM, the CnF, or both. Most importantly, none of the anatomically defined populations expressed an encoding preference for specific task‐related variables. Furthermore, optogenetic activation of SNr neurons projecting to either VL/VM or CnF induced similar behavioral effects in both protocols, and these effects were mainly related to speed control. Altogether, our data suggest that SNr neurons integrate upstream heterogeneous inputs, most likely from direct and indirect pathways of the BG, into speed and position signals that are scaled depending on the behavioral context and broadcasted to different anatomical targets.

Previous reports have demonstrated that the SNr integrates direct and indirect pathway inputs in a competitive way, with the timing of the signals determining whether already commanded movements could be aborted or slowed down [23, 24]. It has also been demonstrated that subpopulations of SNr neurons, when inhibited or activated during striatal optogenetic activation or inactivation, initiate or suppress locomotion bouts, respectively [14, 22]. More recently, it has been shown that the activity of individual SNr neurons exhibits firing pauses or increases linked to precise “granular” representations of forelimb movements [53]. These data support the idea that a competitive relationship between antagonist pathways controls the output nuclei of the BG as an inhibitory gate [54]. Although, in principle, these conclusions may appear to contradict ours, some considerations indicate that the two studies are much less comparable than might initially be assumed. For example, the behavioral conditions differ substantially. While forelimb reaches can, in principle, be compared with the reaches performed in our bimanual coordination task, our protocol also included a fixed holding period of 750 ms, introducing an additional movement phase involving sensory feedback contributions, and we compared the activity of the same neurons across two distinct behavioral protocols. On the other hand, the referred work did not specifically investigate the relationship between neural activity and movement amplitude or speed. For these reasons, our results are more compatible with an expanded view of SNr function, whereby the SNr provides a continuous, moment‐to‐moment modulatory and scalable signal that may be spread to multiple target regions for the modulation of movement speed and position. A signal with such characteristics is consistent with classical views about how the motor cortex represents movement [55, 56, 57]. According to these classical perspectives, motor plans are established as abstract entities and can be performed with different effectors, rather than with fixed torque and angle combinations in specific joints. On the other hand, in a previous work using a bimanual coordination task, we demonstrated that subpopulations of M1 neurons recruit the DLS to determine movement duration [18]. In that context, our data suggest that the SNr would integrate BG upstream signals and contribute abstract representations to modulate speed and position in various behavioral contexts with different spatiotemporal contents.

Previous studies in mice have demonstrated strong linear relationships between SNr spiking activity and the animal's head position during rewarded or unrewarded movements, suggesting a refined Cartesian representation for all movement directions [25]. While those observations are constrained to a “peripersonal” range of movements, our data demonstrate that a similar code is present for complex sequences of movements that require the integration of temporal and spatial information and timed execution (e.g., our spatiotemporal protocol with random changes in treadmill speed from trial to trial). Contrary to reports from the dorsal striatum of rodents [7, 9, 45, 46, 58, 59], which show that 20% to 45% of units express phase‐locked spiking activity in the gait cycle, we found it difficult to identify units with rhythmic autocorrelograms at the same frequency as the cyclic movement of the forelimbs during treadmill locomotion, as only 4.4% of SNr units expressed a similar profile. These rhythmic signals have been traditionally associated with sensory signals that the animals use when walking or running [7, 9, 45, 46], suggesting that the SNr filters low‐level sensory representations and opening the possibility for a broader filtering of different behavioral signals reported for the dorsal region of the striatum.

In the context of the potential anatomical substrates of these SNr signals, a natural question is whether the scaling property is inherited from upstream BG nuclei, for example the striatum, or if it emerges as an exclusive property of the output nuclei. While this is an open question that must be formally addressed experimentally, in our direct antecedents we observed that speed and position signals are reliably found in the dorsolateral region of the striatum in both behavioral protocols [7, 18], indicating that it is possible that scaling properties are not exclusive of the SNr. On the other hand, it has been demonstrated in rodents, primates and felines that SNr neurons project to multiple motor‐related regions, such as the superior colliculus, the motor thalamus, and the mesencephalic locomotor region [12, 38, 39, 60], but different lines of evidence indicate that GABAergic SNr projecting neurons are not a molecularly homogeneous population, but that there may be at least two subpopulations. The most abundant of these subpopulations expresses parvalbumin (PV) [61, 62, 63]. The other is a PV‐negative subpopulation that has been associated with the expression of the vesicular GABA transporter VGAT [63] or glutamate decarboxylase 2, GAD2 [62, 64]. These findings suggest that parallel SNr output projections may be implicated in different functions. For example, Liu and colleagues (2020) show that SNr PV+ and GAD2+ neurons would be more active during periods of high and low motor activity, respectively. Their activation of GAD2+ neurons would also promote transitions to sleep. In our data, we did not separate SNr neurons based on their molecular profile, but rather on their anatomical targets that may be related to locomotion or forelimb movement control. While our data suggest that different SNr neurons would encode similar signals related to speed and body position, future research could complement this approach with transgenic animals to investigate the differential involvement of molecularly defined subclasses of SNr neurons in motor control under different behavioral contexts.

## 4 Methods

All experimental procedures used in this project were approved by the Animal Ethics Committee of the Institute of Neurobiology, National Autonomous University of Mexico (UNAM; Protocols 82.A and 102.A). All procedures conformed to the principles outlined in the Guide for the Care and Use of Laboratory Animals (NIH), and all efforts were made to minimize the number of animals used and their suffering.

### 4.1 Animals

Male Long‐Evans rats (*n* = 6; 250 g–700 g) were housed in pairs at a stable temperature (23°C) and humidity (66%) under a constant 12:12‐h light‐dark cycle (lights on at 8 am). Depending on the experimental phase, animals had free access to food and water, or they were water‐restricted and consumed all the water requirements during the training sessions (20–30 mL in 40–60 min per session,1 session per day). Animal weight was monitored daily and maintained at over 85% of the weight expected by age. If the animals did not consume their daily water proportion, water was provided for short periods after the training. Animals were trained for six days a week with 24 h of free access to water on the seventh day. All experimental procedures were conducted during the light phase of the cycle.

### 4.2 Behavioral Apparatus and Training

#### 4.2.1 Apparatus

For the spatiotemporal task, a commercially available treadmill (NordicTrack T6.1) was customized with Plexiglas walls (50 cm high) to constrain rats to the walkable area on the belt, which is 80 cm long by 20 cm wide. The motor of the treadmill was controlled by a custom‐made program (LabVIEW, National Instruments) and a multifunction computer board (NI USB‐6353, National Instruments). A line of LEDs illuminated the whole apparatus. The front wall of the treadmill was equipped with a liquid well to deliver drops of sucrose solution. A photodetector, positioned 10 cm from the front wall, delimited and signaled each animal's entrances into the designated goal area. A warning signal (1.5 kHz, 65 dB) indicated incorrect early entrances in the stop area (trial errors). For the bimanual coordination task, animals were trained in customized behavioral boxes (50 × 50 × 50 cm) equipped with two sets of levers. A water port and a green LED indicating correct trials were placed between the lever sets. Water rewards were provided with a solenoid valve through the center water port. The lever sets had two white LEDs to indicate their availability. Each set of levers comprised two independent levers protruding 5 cm from the wall. The levers moved vertically and horizontally and were connected to a voltage transducer (3.5 cm = 2.5 V). All training and most experimental procedures reported in this behavioral protocol were performed on the left set of levers. Voltage signals from the levers were digitized and stored at 250 Hz through National Instruments cards (NI PXIe‐6363) and LabView custom‐made routines.

#### 4.2.2 Behavioral Training Spatiotemporal Task

We used a spatiotemporal task as previously described (Rueda‐Orozco and Robbe, 2015; Hidalgo‐Balbuena et al., 2019) with slight modifications. In brief, rats were handled (2 h d^−1^ for 5 days) and familiarized to run on the treadmill at increasing speeds and trained to perform the task. During training, the treadmill speed was fixed at (22 cm s^−1^, 40–160 trials per session, 1 session per day). To determine that the animals had learned the task, a criterion of performance accuracy (≥ 72.5% of correct trials over the last 40 trials, for ≥ 3 consecutive sessions) was established. After at least 30 sessions above the criterion, the speed of the treadmill was increased to 26 cm/s (for at least 30 more sessions) and, finally, to 30 cm/s. After this, the treadmill speed was changed randomly in a range of seven possible speeds (27–33 cm s^−1^) during each trial. Animals were overtrained in this version of the task for at least 30 more sessions before recording. Training was programmed automatically, and the experimenter was not physically present in the room. The position of the animals was determined with a CCD camera (acA640‐120fc, Basler, 100 frames s^−1^, 9 pixels cm^−1^) positioned next to the treadmill, and a fluorescent marker was attached to the left forelimb. The marker's positions or the center of the animal's body was automatically extracted with a custom‐made program (Vision, National Instruments) and averaged in 400 ms‐long sliding windows (the average duration of a step cycle). To quantify the behavioral stereotypy, we extracted the position and speed time‐courses from every trial of every session. Position (or speed) trajectories were aligned to the entrance times (i.e., at the end of movement sequences), and Pearson's correlation coefficients were computed for all the possible pairs of trials. For speed, we restricted the analysis to the last 2.5 s before the entrance time.

#### 4.2.3 Bimanual Coordination Task

We used a bimanual coordination task as previously described (Báez–Cordero et al., 2020; Pimentel–Farfan et al., 2022). Animals were trained to hold the two levers, one with each paw, and to vertically displace them at least 2.6 cm (vertical threshold) for a fixed time threshold (50, 500, 750 ms) (Figure 1b). The time counter started as soon as both levers crossed the spatial threshold and was restarted when either lever was above the threshold. Trials were self‐initiated; there was no time limit to start a trial or obtain a reward. A white light over the levers indicated that the set of levers was available. Correct trials and availability of the reward were indicated with a green light (on for 1 s) over the water port. During this time, the white light over the levers was turned off (800 ms), and the animal could not get another reward; after that time, the white light indicated that the pair of levers was available. The animal performed an unlimited number of trials (∼250 trials) over the lapse of 40 min to 1 h. *Training*. Rats were exposed to the training boxes for one session (40 min) before the beginning of training and the water restriction. During the next session, the rats were trained to obtain rewards (drops of water, 60 µL) when approaching the water port. In subsequent sessions, animals were trained to obtain rewards by touching any lever and slightly pressing it down (> 0.1 cm). In general, all animals quickly learned the rule of touch and could displace any lever in less than two sessions. Then, animals learned to displace both levers simultaneously. During the first 8 to 15 sessions, we progressively increased the spatial threshold to get a reward until the final target position was reached. In the same sessions, the temporal threshold was established at 50 ms and progressively increased to 750 ms.

### 4.3 Implantation of Silicon Probes for Unit Recording in Behaving Animals

Six animals were used for freely moving experiments. Under deep sevoflurane anesthesia, 64‐channel silicon probes (Buzsaki‐64‐L, Neuronexus) were implanted approximately 500 micrometers above the SNr (coordinates: AP = −5.4; ML = 2.4). Two miniature screws were implanted in the bone above the left cerebellum and parietal cortex and served as ground and reference. The microdrive and the connector were secured to the skull with miniature screws and two types of adhesive cement (C&B Metabond and Meliodent). To ensure long‐term recording stability, the implants were mechanically protected by a miniature copper mesh that served both as a compact Faraday cage, reducing electrical noise, and as a rigid protective barrier that prevents direct impacts on the microdrive assembly. After surgery, a prophylactic analgesic treatment was administered (Meloxicam, 0.5 mg/kg). After the recovery period (at least 6 days), probes were slowly lowered toward the recording sites (75–150 mm per day). Recordings were performed at depths between 6.4 and 8.5 mm below the surface of the brain. During recording periods and to maximize the probability of recording different neuronal populations across sessions, electrodes were advanced by 50–100 µm between sessions, and recordings were never initiated until the electrodes had stabilized for at least 6 h following any adjustment.

The first three animals were recorded sequentially in both tasks. First, they were recorded in the spatiotemporal task (between 19 and 25 sessions). After this period, the animals were recorded and trained in the bimanual coordination task. Another group of four animals was first trained in both tasks simultaneously. On each day, the animal was first trained in the spatiotemporal task, and immediately after its last trial, it was transferred to the behavioral chamber for training in the bimanual coordination task. After the animals reached proficient performance in both tasks, they were subjected to surgical procedures for microelectrode array and optic fiber implantation and viral infections. Ten days after the surgery, the animals resumed training in both tasks while being recorded.

### 4.4 Electrophysiological Data Acquisition and Processing

Wide‐band (0.1–8000 Hz) neurophysiological signals from silicon probes (Neuronexus, Buzsaki‐64L) were amplified 1000 times via the Intan RHD2000‐series Amplifier evaluation system or Plexon VLSI head stages and a PBX2 amplifier and continuously acquired at 20 kHz on two synchronized National Instruments A/D cards (PXi‐e 6363, 16‐bit resolution). Spike sorting was performed using a semi‐automatic pipeline based on KlustaKwik (http://klustakwik.sourceforge.net) [65], followed by manual curation using the graphical spike sorting application Klusters (http://klusters.sourceforge.net) [66]. During this final curation step, multiple quality metrics ‐including waveform stability over time, autocorrelograms, cross‐correlograms, and evidence of waveform drift‐ were visually inspected to identify and exclude units potentially affected by electrode movement or electrical artifacts.

### 4.5 Nueral Data Analysis

#### 4.5.1 Spiking Activity During Spatiotemporal Task Execution

First, spiking activity was extracted from the running periods of the task (i.e., treadmill onset to the end of movement sequences). Inter‐trials were not analyzed. Trials were divided into 100 ms none‐overlapping windows, and the firing rate was calculated for each window (spike count divided by 0.25) and smoothed with a Gaussian kernel filter with a standard deviation of 200 ms. The position of the center of mass of the animals or a fluorescent marker on the head or forelimb contralateral to the recording site was extracted from video recordings at 100 fps. Then, Pearson's partial and multiple correlation coefficients between spiking activity and different parameters of displacement were calculated for each cell. *Spiking activity during bilateral coordination task execution*. For the activity in raster plots and color‐coded matrices in Figure 3, first we extracted spiking activity from each trial in 1 ms bins over an 8 s window centered on reward onset (± 4 s). Spike trains from each trial were smoothed with a Gaussian kernel filter with a standard deviation of 50 ms. For each neuron, the average peri‐event histogram was transformed into Z‐scores. To calculate kinematic and lever press‐related correlations, trials (± 4 s centered on reward onset) were divided into non‐overlapping windows of 100 ms. The firing rate was calculated for each window (spike count divided by 0.1) and smoothed with a Gaussian kernel filter with a standard deviation of 200 ms. The position of the animal's head was automatically extracted from 30 fps videos with a custom‐made program (Vision, National Instruments) and averaged in 100 ms‐long sliding windows. The acceleration (3 axes) of the head movements was extracted directly (20 kHz) from the accelerometers integrated to recording head stages (Intan Technologies RHD2132). The movement trajectories from the levers were obtained as described in the previous sections. Pearson's partial and multiple correlation coefficients between spiking activity and the position, speed, and acceleration of the head and levers were calculated for each cell.

#### 4.5.2 Pattern Classification Method

A PCA/Silhouette‐based method was used as reported in previous works [18, 41, 43, 47] with appropriate modifications. Here, PCA was applied to: (1) the average spike wave shapes of neurons depicted in Figure S4 and (2) the z‐scored perievent histograms of the spiking activity during behavioral execution in the spatiotemporal task (activity was aligned to the treadmill onset and time normalized between treadmill onset and the end of the sequence of movements) and the bimanual coordination task (8 s activity centered at reward onset), both depicted in Figure 7. To assign cells to specific clusters with similar characteristics, k‐means was applied to the first three principal components (PCs). To obtain the best classification, the process was repeated 1000 times with projections ranging from 1 to 12 clusters. Each projection was scored with the Silhouette method, and the projections with the highest Silhouette scores were selected.

#### 4.5.3 Statistics

The electrophysiological and behavioral data are presented as median + 25th and 75th percentiles. For all groups, normality was estimated with the Kolmogorov–Smirnov test. Statistical significance for group comparison was calculated using the Mann–Whitney or Kruskal‐Wallis test for electrophysiological and behavioral data. Multiple comparison analysis was performed with the Bonferroni post hoc test. Statistical analysis was performed using MATLAB software (The MathWorks, Inc.). Differences were considered statistically significant if the *p*‐value was ≤ 0.05.

## Author Contributions

Conceptualization: A.S.B.C., P.R.O.; methodology: A.S.B.C., P.R.O.; investigation: A.S.B.C., D.I.O.R., P.R.O.; data curation: A.S.B.C., P.R.O.; formal analysis: A.S.B.C., P.R.O.; Writing – original draft: A.S.B.C., P.R.O.; Writing – review and editing: A.S.B.C., P.R.O.; supervision: P.R.O.; project administration: P.R.O., C.I.P.D.; funding acquisition: P.R.O.

## Funding

This work was funded by grants: UNAM‐DGAPA‐PAPIIT: IN200822; IG200424 to PRO; SECIHTI (formerly CONAHCYT): FDC‐2016‐1702, CF‐2023‐I‐7 to PRO. This work was supported by Programa de Apoyos para la Superación del Personal Académico from UNAM (PASPA‐DGAPA‐UNAM 2024) to PRO.

## Conflicts of Interest

The authors declare no conflicts of interest.

## Supporting information




**Supporting File**: advs77621‐sup‐0001‐SuppMat.docx.

## Data Availability

The data that support the findings of this study are available from the corresponding author upon reasonable request.
