# Supplementary material for: Abstract Representation of Movement Parameters in Basal Ganglia Output
Source: Adv Sci (Weinh). 2026 Sep 14:e77621. Online ahead of print. doi: 10.1002/advs.77621 (PMC13573267; doi:10.1002/advs.77621)
Supplement: Supplementary file 1 [file ADVS-9999-e77621-s001.docx]

**Abstract representation of movement parameters in basal ganglia output**

Ana Silvia Báez-Cordero*, Diana I. Ortega-Romero, Claudia I. Pérez-Díaz, Pavel E. Rueda-Orozco*.

Department of Neurobiology of Development and Neurophysiology, Institute of Neurobiology, UNAM

*Corresponding author: [anasbaez@gmail.com](mailto:anasbaez@gmail.com); [pavel.rueda@gmail.com](mailto:ruedap@unam.mx)

**Supplementary Figures**

**Figure S1.** Examples of error trials. a) Two error trials (indicated by circled numbers) taken from the four neurons depicted in Figure 2. For each neuron and trial position (top), speed (middle), firing rate (bottom) are shown. Firing rate is also displayed as blue trajectories in the corresponding position or speed panels depending on the strength of the correlations depicted in Fig. 2 (for negative correlations the firing rate trace is inverted).

**Figure S2**. The SNr integrates behavioral variables in both behavioral protocols. Median of Akaike information criterion (AIC) values computed for all recorded units using regression models with all possible combinations of variables for neurons recorded in the treadmill spatiotemporal task (a) or the bimanual coordination task (b). Red dotted line indicates the AIC median value of the best model.

**Figure S3.** Scaling code in the SNr neurons with the highest correlation values. Firing rate range in the spatiotemporal and bimanual coordination tasks (color coded) for neurons classified as position (a) or speed related (b). Same as in a,b, but for position (c) or speed (d) content by unit of frequency. Range of position (e) or speed (f) expressed during behavioral sessions. Data correspond to neurons that expressed partial correlation coefficients for speed or position above de 99.5 percentile of the surrogated distribution. The central line and box in boxplots represent the median and 25^th^-75^th^ percentiles, and whiskers extend to the most extreme datapoints excluding outliers. *** p < 0.001, two-sided Wilcoxon rank sum test.

**Figure S4.** Spike waveform classification of SNr neurons. (a) Silhouette values from 1,000 iterations of k-means clustering (k = 2–10) applied to the PCA projections of the average spike waveforms from neurons recorded during movement execution. (b) Three-dimensional scatter plot showing the first three principal components of the spike waveforms for each neuron. The color code in (b–f) corresponds to the optimal two-cluster solution identified in (a). (c) Average spike waveforms (solid lines) for neurons classified into the two groups based on their spike waveform. (d) Firing rates of neurons belonging to each group. Number (bars) and percentage (indicated on each bar) of SNr neurons exhibiting short-latency responses to VL/VM, CnF, both (color coded as in Figure 6l) for neurons belonging to group 1 (e) and group 2 (f). Partial correlation coefficients during behavioral execution in the spatiotemporal task (g; K-W, *df = 7, X^2^ = 481.36, p < 0.001*) and the bimanual coordination task (h; K-W, *df = 9, X^2^ = 388.77, p < 0.001*).

**Figure S5.** Execution variables encoded in neurons projecting to the VL/VM, CnF, or Both. Histograms displaying population partial and multiple correlation coefficients (absolute values) between firing rate and task variables during treadmill (a) and bimanual task (b) execution. Gray distributions represent partial and multiple correlation coefficients obtained with surrogated spike trains from the same neurons.

**Figure S6.** SNr population dynamics. Z-scored average firing rates of neurons recorded during behavioral execution in the treadmill-based spatiotemporal protocol (a) and the bimanual coordination task (b). Neurons (rows) are sorted by the time of their average peak firing rate during complete treadmill runs (a) or between -2 s and reward delivery (time 0; green arrow and white dotted line) in the bimanual coordination task (b). In each panel, the average position and speed trajectories (white traces) across animals and sessions are shown for reference. In (a), neural activity was aligned to treadmill onset (time 0), and the interval between treadmill onset and reward delivery (green arrow and white dotted line, time 1) was normalized. Explained variance of the first five principal components (PCs) derived from population dynamics during behavioral execution in the treadmill (c) and bimanual coordination (d) protocols. Low-dimensional PC1 (e, f), PC2 (g, h), and PC3 (i, j) projections of the population activity, together with the correlation coefficients (box plots on the right) between each PC trajectory and three relevant behavioral variables over time, are shown for neural activity recorded during behavioral execution in the treadmill (e, g, i) and bimanual coordination (f, h, j) protocols. Colored and gray box plots represent observed and surrogate activity from the same neurons, respectively. Box plots indicate the median and the 25th and 75th percentiles. Statistical differences were assessed with K-W and Bonferroni post hoc test, P < 0.05: e) df = 5; X2 = 408.45; f) df = 5; X2 = 445.63; p < 0.001; g) df = 5; X2 = 369.94; h) df = 5; X2 = 324.91; p < 0.001; i) df = 5; X2 = 321.06; j) df = 5; X2 = 163.01; p < 0.001.

**Figure S7.** Behavioral examples of targeted SNr neurons projecting to the VL/VM and CnF. a) From left to right, for one animal injected in VL/VM (blue code), and two animals injected in the CnF (purple code); behavioral variables for stimulated and non-stimulated trials during execution in the spatiotemporal protocol: sorted sequence durations for all non-stimulated and stimulated trials; median (solid lines) and 25^th^ and 75^th^ percentiles (dashed areas) for sequence duration, position and maximum speed displayed at different treadmill speeds; average position (top) and speed (bottom) trajectories for all stimulated and non-stimulated trials. For the same animals as in a) but for variables collected in the bilateral coordination protocol: from left to right, interlimb correlations, onset synchrony, overshoot and maximum speed for non-stimulated (black code for the rest of the figure) and stimulated trials (colored code, for the rest of the figure). Box plots indicate median and 25^th^ and 75^th^ percentiles for all trials from all stimulated sessions; pairs of dots united by lines indicate medians of each session.

**Graphical abstract**

The basal ganglia output provides a scalable velocity/position signal across behavioral contexts, supporting the idea that these variables are represented in the brain as abstract entities rather than as fixed changes in joint angles or specific sets of body effectors.
